# Electrocatalytic performance of NiNH_2_BDC MOF based composites with rGO for methanol oxidation reaction

**DOI:** 10.1038/s41598-021-92660-8

**Published:** 2021-06-28

**Authors:** Lubna Yaqoob, Tayyaba Noor, Naseem Iqbal, Habib Nasir, Asad Mumtaz

**Affiliations:** 1 School of Natural Sciences (SNS), National University of Sciences and Technology (NUST), Islamabad, Pakistan; 2 School of Chemical and Materials Engineering (SCME), National University of Sciences and Technology (NUST), Islamabad, Pakistan; 3 U.S-Pakistan Center for Advanced Studies in Energy (USPCAS-E), National University of Sciences and Technology (NUST), H-12 Campus, Islamabad, 44000 Pakistan

**Keywords:** Chemistry, Energy science and technology, Materials science, Nanoscience and technology

## Abstract

Present work comprehensively investigated the electrochemical response of Nickel-2 Aminoterephthalic acid Metal–Organic Framework (NiNH_2_BDC) and its reduced graphitic carbon (rGO) based hybrids for methanol (CH_3_OH) oxidation reaction (MOR) in an alkaline environment. In a thorough analysis of a solvothermally synthesized Metal–Organic Frameworks (MOFs) and its reduced graphitic carbon-based hybrids, functional groups detection was performed by FTIR, the morphological study by SEM, crystal structure analysis via XRD, and elemental analysis through XPS while electrochemical testing was accomplished by Chronoamperometry (CA), Cyclic Voltametric method (CV), Electrochemically Active Surface Area (EASA), Tafel slope (b), Electron Impedance Spectroscopy (EIS), Mass Activity, and roughness factor. Among all the fabricated composites, NiNH_2_BDC MOF/5 wt% rGO hybrid by possessing an auspicious current density (j) of 267.7 mA/cm^2^ at 0.699 V (vs Hg/HgO), a Tafel slope value of 60.8 mV dec^−1^, EASA value of 15.7 cm^2^, and by exhibiting resistance of 13.26 Ω in a 3 M CH_3_OH/1 M NaOH solution displays grander electrocatalytic activity as compared to state-of-the-art platinum-based electrocatalysts.

## Introduction

At present, to fulfill the emergent worldwide energy demands and to replace the non-renewable energy sources, the invention and development of renewable, green, and economical energy sources is the field of attention and investigation for scientists and researchers^1–5^. Amongst the Fuel Cells, Direct Methanol Fuel Cell (DMFC) is the prospective candidate for handy devices and light-duty vehicles owing to prerequisite low temperature requirement, liquid nature of the fuel, quick refueling, eminent power density, facile charging, minimum environmental influence, and high yield synthesis of H_2_^6–16^.

The complete oxidation process with *E*^0^ values versus RHE is represented as follows:^17^

$${\text{CH}}_{3} {\text{OH}}~ + ~3/2{\text{O}}_{{2~}} \to {\text{CO}}_{{2~}} + 2{\text{H}}_{2} {\text{O}}\quad E^{0} = 1.213\;{\text{V}}\quad \left( {{\text{Overall}}\;{\text{reaction}}} \right).$$

The MOR proceeds identically in an acidic and basic media except that CO to CO_2_ conversion occurs by OH^–^ group (provided by alkali) very smoothly in basic media while the same conversion takes place after water dissociation in an acidic environment with a low reaction rate^18^. To overcome the problems of (a) depressed redox process, (b) high manufacturing cost, (c) catalyst inactivation by reaction intermediates, and (d) sluggish kinetics, the development of a most appropriate and low-cost electrocatalyst with (i) flexible morphology, (ii) prompt electrons, ions, and reaction products transport (iii) The strong interaction between catalyst and reactants with adequate contact area, and (iv) upright inherent activity are the requirements of current research to replace highly active but expensive and less stable Pt metal-based catalysts^19–21^. Currently, contrary to noble metals, transition metal-based oxides, carbides, nitrides, borides, as well as double layered hydroxides (LDH) are embryonic materials with a tremendous performance for the MOR^22–25^. Additionally, the transition metal-based coordination polymers (MOFs) based electrocatalysts are the area of interest for researchers due to their less density, gigantic surface area, porosity, tunable, and stable nature^26–28^.

Moreover, in nickel-based MOFs (a) easy access to economical precursors (b) variable valency of Ni metal (+ 1 to + 3), predominantly higher oxidation state (c) formation of nickel oxides and hydroxides on the surface, and (d) conversion of Ni(OH)_2_ (β) → NiOOH (β) → NiOOH (γ) during electrochemical testing all are responsible for the excellent electrocatalytic response. Moreover, the inclusion of mesoporous, conductive supports (GO, rGO, Graphene, CNTs) is an important strategy to promote catalyst stability and charge transfer proficiency by the development of new pores and control over pore size which in turn enhances the surface area and leads to improved electrocatalytic activity^29–31^.

In 2018, Li et al. reported highly active spongy nickel frameworks deposited metal Pt-Ni nanoparticles with improved MOR activity and stability due to high CO tolerance, great surface area, and effective utilization of active sites. The Pt-Ni/C electrocatalyst was prepared by Ortega and colleagues in the same year where the presence of double metals, reduction in particle size of Pt due to Ni inclusion, huge surface area, the formation of metal oxides and hydroxides, and reformed electronic structure due to synergistic effect leads to excellent MOR activity while Pt Nanostructured Carbon (Pt/NC) composite prepared by Ferrer and collaboraters show greater current density than Pt/rGO and Pt/C due to enhanced electron transport at the electrode–electrolyte interface along with hierarchal structure^32–34^.

GCE deposited Ni (OH)_2_, fabricated by a potentiostat method was tested by Raoof et al*.* in 2013 for MOR. In the redox process, the first step is the oxidation of Ni (OH)_2_ to NiOOH and the second step is the reduction of NiOOH to Ni (OH)_2_ by methanol. The MOR is governed by the diffusion process at low concentrations whereas it is controlled by the electrocatalytic reaction between Ni (III) and methanol at comparatively higher concentrations while in 2014, nanocubes of Co_3_O_4_ / 2 wt% rGO composite with a current density of 0.37 mA/cm^2^ at 0.8 V versus RHE along with good stability during the MOR were presented by Mehmood et al.^35,36^.

Stephanie and colleagues in 2016 fabricated Fe–Ni NPs as a MOR catalyst. During the reaction, the Fe (core)-Ni (shell) nanoparticles show the oxidation of Ni shell to α-Ni(OH)_2_ and β-NiOOH, and the formation of metallic nanoparticles as well. The tested sample provides the forward current density of 0.048 A/cm^2^ at an anodic potential of 1.58 V vs RHE and current density rises with an increase of methanol concentration^37^.

In 2018, Surfactant assisted co-reduction approach was instigated by Li et al*.* to formulate bimetallic Ni–Sn nanoparticles (3–5 nm). Carbon black supported Ni–Sn NPs due to (a) modified electronic structure (b) inhibited nickel surface poising effect due to oxidation of side product to CH_3_OH, and (c) weak binding of reaction intermediates delivers the currents density of 0.05 A/cm^2^ at 1660 mV and show stable current response with little variation till 5000 s^38^. In 2019, solvothermally synthesized NiO-MOF@rGO composites were reported by Noor et al. where optimized NiO-MOF/5 wt% rGO composite show good electrocatalytic activity by possessing low resistance and Tafel slope along with high diffusion coefficient via minimum loss of originally attained current density even after 60 min while in the same year a novel nickel-based anode catalyst (Ni_9_S_8_-C) was fabricated by Hussain and associates. The resultant, Ni_9_S_8_ delivers the current density of 0.052 A cm^−2^ with little loss of current density even after 5000 s as well as minimum resistance because of strong binding between nanoparticles and carbon substrate^23,39^.

Yaqoob et al. in 2019 fabricated Ni-BTC MOF/1–5 wt% rGO composites for MOR through a solvothermal technique. The hexagonal shape Ni-BTC/4 wt% rGO composite with high current density, minimum Tafel slope and capacitance, and long-term stability show a good response towards MOR. Furthermore, the product analysis by NMR confirms the oxidation of methanol to formic acid with 62% conversion efficiency^40^. In the same year, a series of spindle-shaped bimetallic MOFs (Fe-Co NH_2_BDC) with a variable Fe/Co ratio was successfully synthesized via a one-pot solvothermal approach by Bushra et al*.* In an alkaline medium, the best material (Fe/Co = 1) with high porosity and synergetic effect between MOF and two metals results in low overpotential, high Tafel slope, and long term stability for 6 h during the OER process^41^.

In this study, a solvothermally fabricated NiNH_2_BDC MOF/1, 2, 5 wt% reduced form of graphene oxides hybrids (NiNH_2_BDC/rGO) were thoroughly analyzed for the CH_3_OH oxidation process in 1 M NaOH/3 M CH_3_OH solution. In MOF/rGO composites diverse aspects such as; (a) availability of manifold Ni oxidation states; + 2 to + 3, smoothened redox process via facile electron movement (b) coordination compound formation propensity of nickel (c) development of defects and vacancies (d) rGO sheets tremendous stability and conductivity (e) escalated surface area of MOF (f) MOF NPs homogenous dispersal on the surface of the support, and (g) synergistic effect between rGO and MOF, all support the incredible enactment of as-synthesized materials for the methanol oxidation reaction. According to our literature survey, the catalytic tendency of NiNH_2_BDC MOF/1, 2, 5 wt% reduced graphene oxide hybrids for the CH_3_OH oxidation process has not been conveyed in the literature to date.

## Experimental section

The 99% pure reagents and chemicals without further treatment were utilized for synthesis. Prerequisite chemicals for the fabrication purpose include Ni (NO_3_)_2_.6H_2_O, 2-Aminoterephthalic acid (NH_2_BDC), potassium permanganate (KMnO_4_), sodium nitrate (NaNO_3_) which were procured from Sigma Aldrich while Dimethylformamide (DMF), hydrazine hydrate, hydrogen peroxide, and sulphuric acid were picked up from Merck.

### Fabrication of Ni NH_2_BDC MOF

Synthesis of NiNH_2_BDC MOF was carried out by an already described solvothermal scheme after slight modification^42^. 1 mol solution of both Ni (NO_3_)_2_.6H_2_O and 2-Aminoterephthalic acid in 15 ml DMF were prepared simply by stirring with subsequent slow mixing of nickel nitrate clear solution in linker solution. The homogenous mixture of MOF precursors acquired after one hour of stirring was drizzled to a 23 ml capacity autoclave (Teflon lined) and retained for twenty-four hours at 393 K in an electric oven. After reaction accomplishment, the room temperature was attained by slow cooling of the autoclave. Light green powdered material was achieved after repetitive washing of autoclave product with DMF and ethanol via centrifugation and vacuum drying at 348 K for 24 h.

### Fabrication of GO and rGO

Reported Hummer’s method was utilized for GO Synthesis while rGO was synthesized through reduction of GO by hydrazine hydrate via 24 h reflux at 373 K^43,44^.

### Fabrication of Ni NH_2_BDC MOF/rGO composites

**Figure 1:**
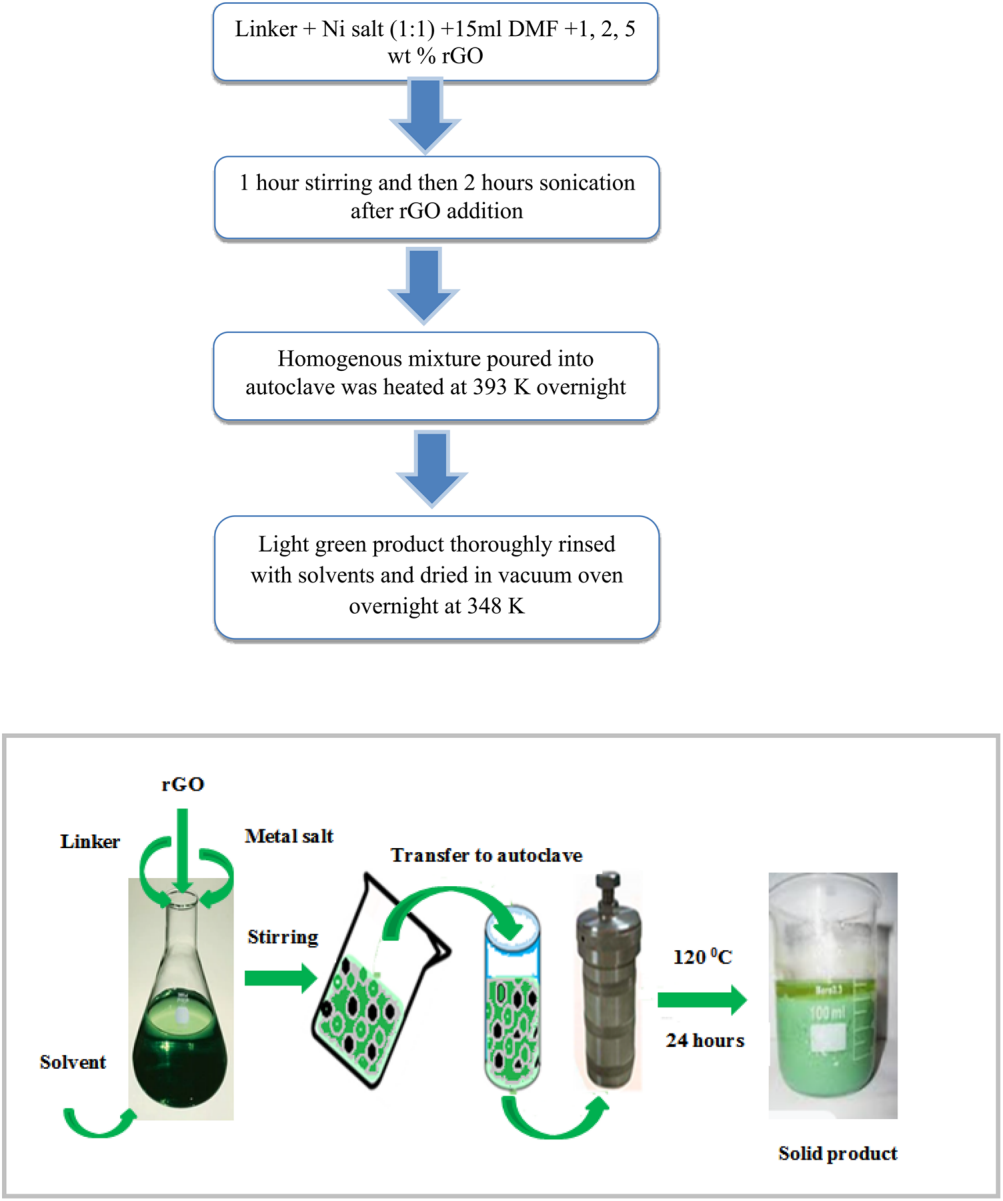
Stepwise synthetic scheme of NiNH_2_BDC MOF/1, 2, 5 wt% reduced graphitic carbon hybrids and flow sheet diagram for the synthesis of NiNH_2_BDC MOF/1, 2, 5 wt% reduced graphitic carbon hybrids.

A NiNH_2_BDC MOF/rGO hybrids (MOR catalyst) series was prepared by following the above-stated procedure. A clear solution of MOF obtained by the above-mentioned scheme and appropriate amount rGO was sonicated for two hours to acquire a uniform blend and then transmitted to stainless steel autoclave with Teflon inner cavity for 24 h reaction at 393 K. A dry solid product was obtained after 3–4 times washing with DMF/ethanol via centrifugation followed by 24 h vacuum drying at 348 K (Fig. 1).

### Materials characterization

For comprehensive characterization of as-synthesized samples, a Scanning Electron Microscope (VEGA 3 TESCAN) was employed for exterior structure and morphology analysis. X-ray Powder Diffractometry (STOE Germany) was implemented for phase purity and crystalline nature scrutinization (Kα = Cu radiation, 0.1.54 ^0^A, scanning range = 5°–80°, step size = 4°/s at 5 mA and 20 kV) while validation of functional groups and metal–ligand strong interaction was corroborated through FTIR Spectrophotometer (Perkin spectrum) by selecting wavenumber range of 500–4000 cm^-1^. Binding energy and composition records were collected by XPS (MI-600) respectively. The degree of defects and graphitization, as well as the extent of crystallinity, was ascertained via Raman Spectroscopy, and stability of material was observed through Thermo gravimetric analyses (TGA) by using a thermo-gravimetric analyzer (Perkin Elmer Pyris 1, Champaign, IL, USA). The temperature was increased from 20 to 500 °C at a heating rate (5 °C min^−1^) under an airflow rate of 20 ml min^−1^.

### Electrocatalytic measurements

An inclusive electrochemical evaluation was done in 3 M CH_3_OH/1 M NaOH mixture through the Gamry apparatus (Ref 3000/3000 AE). To get homogenous ink of electrocatalyst, 2.0 mg of sample (0.85 mg/cm^2^), 97 µl ethanol, and 3 µl Nafion were sonicated for 40 min, and then 0.003 ml of electrocatalyst ink was plunged on a glassy carbon electrode (GCE = working electrode) by micropipette. Pt wire and Hg/HgO were chosen as auxiliary and reference electrodes correspondingly. The selected voltage window for electrocatalytic response through Cyclic Voltammetry (CV) and stability testing for 3600 s via chronoamperometry at a fixed potential of 0.69 V was − 0.1–0.7 V (vs Hg/HgO). Moreover, the frequency range of 1–1 × 10^5^ Hz was picked to find out system resistance at an amplitude of 0.005 V.

## Results and discussion

NiNH_2_BDC MOF/1,2,5 wt% rGO hybrids were systematically evaluated through XPS, FTIR, XRD, SEM, Cyclic Voltammetry, Tafel slope, Electrochemical Impedance Spectroscopy, EASA, Mass Activity, roughness factor, and Chronoamperometry.

**Figure 2:**
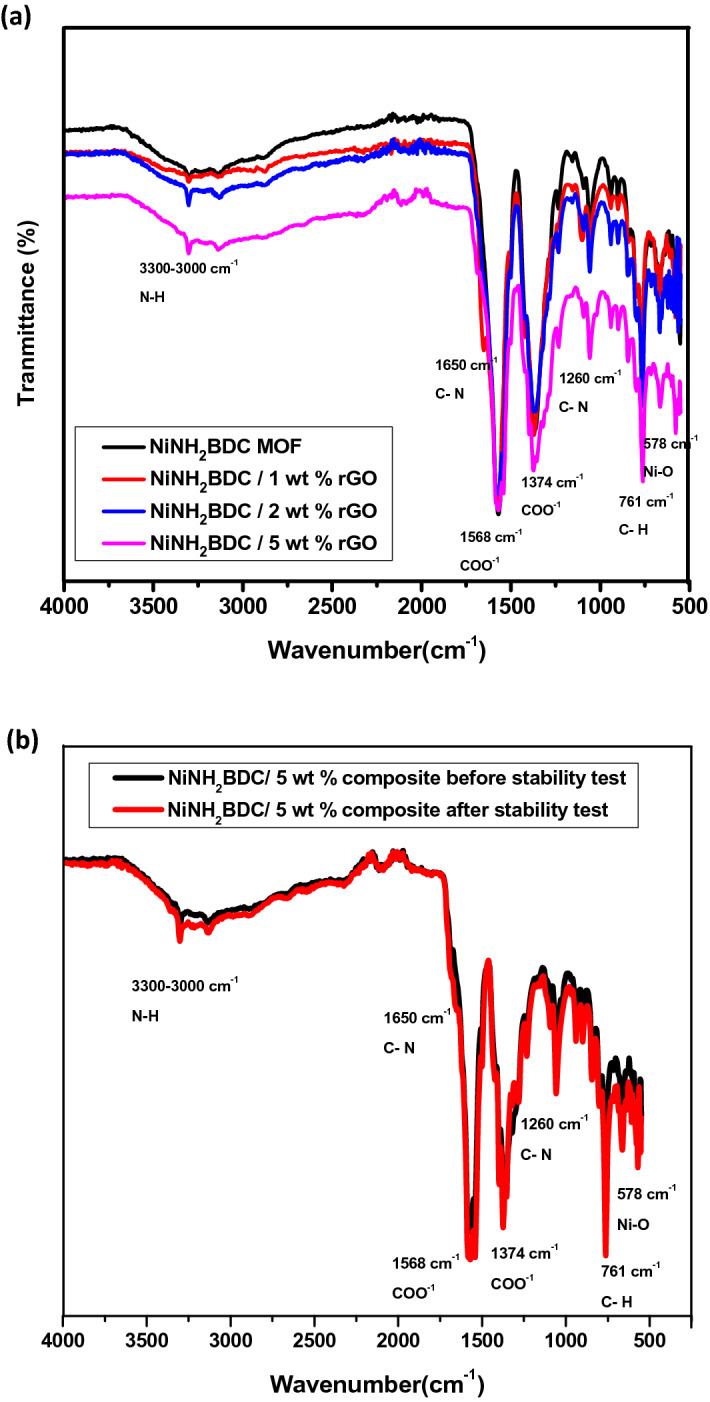
(**a**) Fourier transformed infrared spectrum of NiNH_2_BDC MOF/1, 2, 5 wt% reduced graphitic carbon hybrids (**b**) FTIR spectra of NiNH_2_BDC/5 wt% rGO composite before and after stability test.

In the FTIR spectrum of fabricated samples (Fig. 2a) the COO^−1^ group symmetric and asymmetric stretching vibrations generate resilient adsorption bands at the position of 1568 and 1374 cm^−1^ and the gap between these two bands designates the connection of the COO^−1^ group of the linker with nickel-metal through the bidentate mode of linking. The band at 1655 cm^−1^ besides 1250 cm^−1^ indicates the N–C group stretching mode of vibration as well as divulges the coordinated DMF group manifestation while NH_2_ group stretching and bending vibrations bands appear at 3309 and 1684 cm^−1^, respectively^45–48^. The region between 3300 and 3000 cm^−1^ was occupied by asymmetric & symmetric vibration bands of the H–N group^49^. C–H peak appears at 754 cm^−1^ while the evident peak at 587 cm^−1^ approves the presence of nickel and its coordination with the COOH group oxygen. Figure 2b represents the comparison of FTIR spectra of NiNH2BDC/5 wt% rGO composite before and after the stability test^50,51^.

**Figure 3:**
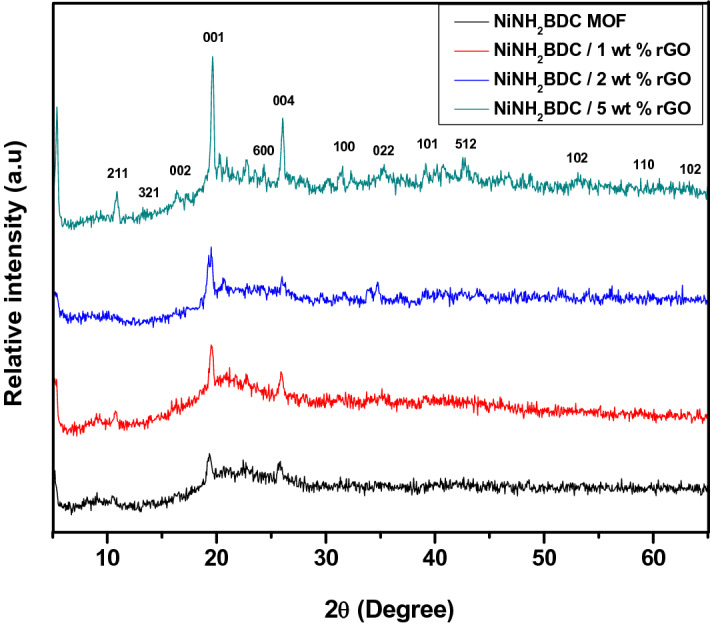
X-ray diffraction pattern of NiNH_2_BDC MOF/1, 2, 5 wt% reduced graphitic carbon hybrids.

In the reported samples XRD pattern (Fig. 3), less significant peaks within 2θ range of 42°–52° correspond to the Ni NH_2_BDC MOF distinctive peaks, while less intense peaks at 15°, 25°, 35°, 38°, 40°, and 61° ensure the development of Ni (OH)_2_ during the reaction accompanied with DMF peak at 2θ, 7.5° and attributed to restricted hydrogen bonding leading to swelling effect and establishment of MOF pores (JCPDS. No:02-1216)^50,52^. The rGO characteristics peaks are positioned at 17° and 23° and the gradual incline in peak intensity from MOF to NiNH_2_BDC/5 wt% rGO composite reflects the successful inclusion of rGO and composites synthesis. The XRD pattern closely resembles the literature data^53,54^.

The particle size, crystalline shape, and morphology appraised at different magnifications via SEM study display the presence of hexagonal shape particles while Reduced Graphene Oxide sheets working as a MOF support not only control the size of MOF nanoparticles but also helps in a MOF NPs fine dispersal on its panes and prevents the agglomeration. During the optimization of the synthetic conditions, it was observed that the addition of a small amount of PVP further controls the growth, shape, and size of hexagonal particles. Furthermore in EDX analysis for elemental composition, the increase in carbon content from MOF towards 5 wt% rGO composite validates the successful synthesis of composites (S.I)^50,55^.

**Figure 4:**
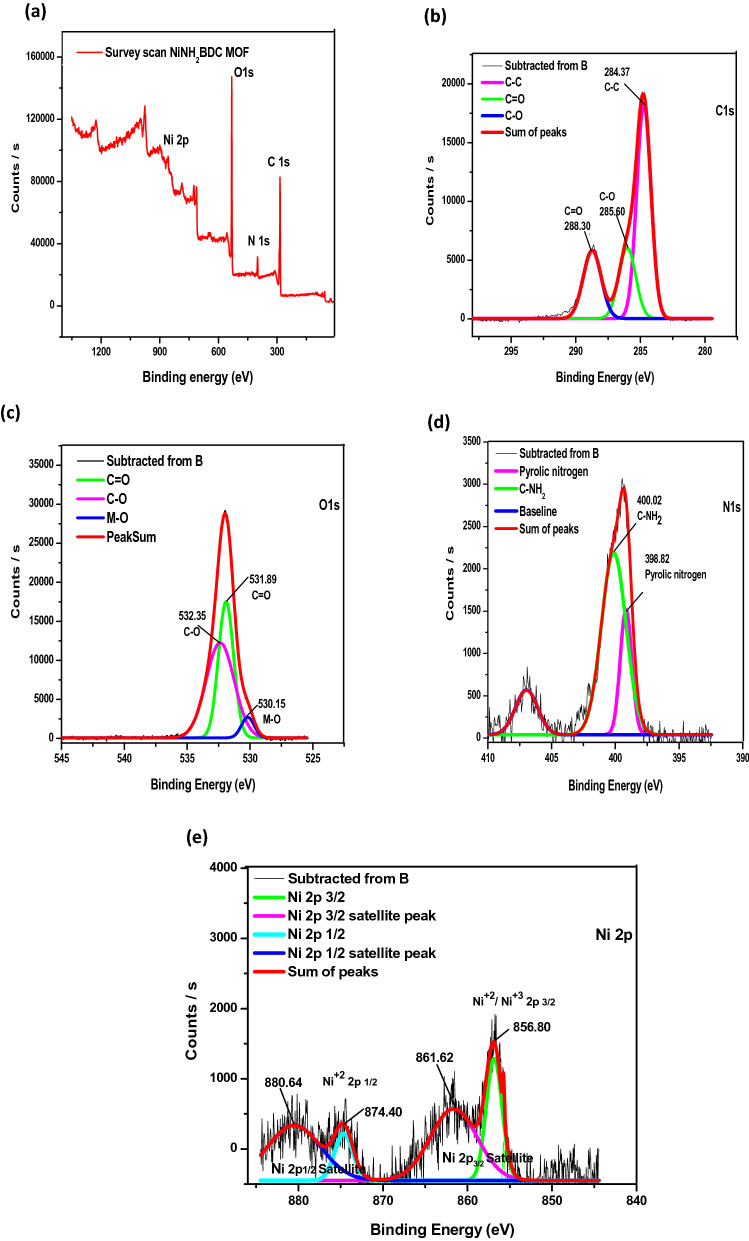
X-ray photoelectron spectra of NiNH_2_BDC (**a**) Survey scan (**b**) Carbon (**c**) Oxygen (**d**) Nitrogen, and (**e**) Nickel.

The information about elemental composition, binding energy, and metal oxidation state was obtained by XPS (Fig. 4). The C 1 s spectrum comprises 3 peaks at B.E (binding energy) value of 284, 286, and 288 eV stipulate the existence of C–C, C-O, and C = O groups, respectively while oxygen spectra deconvolution in 3 peaks with a binding energy value of 530, 531, and 532 eV specifies the existence of M–O, C = O, and C-O. Fragmentation of nitrogen XPS spectra into two peaks of 402 and 403 eV represents the presence of the amine and pyrrolic nitrogen, respectively^56^. In the nickel spectrum, the presence of Ni^+2^/Ni^+3^ was verified by the peaks in the range of 853–875 eV. The peaks at 871.8 eV for Ni 2p_1/2_ and 853.1 eV for Ni 2p_3/2_ indicate the spin-orbital coupling of Ni (+ 2) while peaks positioned at binding energy value of 874.73 eV (Ni 2p_1/2_) and 856.80 eV (Ni 2p_3/2_) are due to spin-orbital coupling of Ni (+ 3). The energy difference of 17.6 eV not only confirms the existence of Ni^+2^/Ni^+3^ but also supports the nickel hydroxide manifestation^55,57–62^.

**Figure 5:**
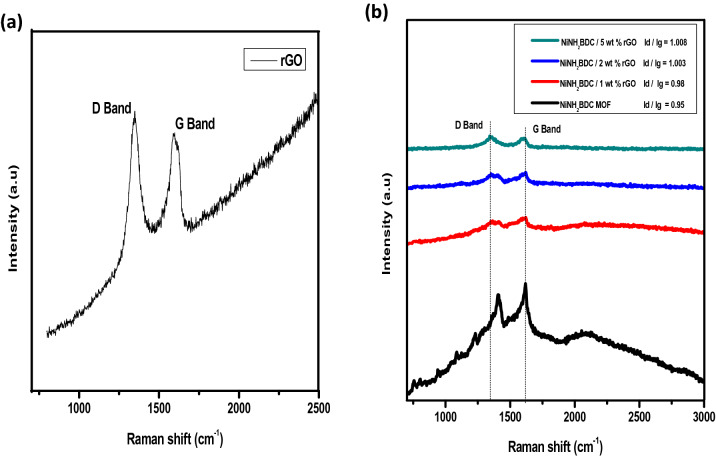
Raman spectra of (**a**) rGO (**b**) NiNH_2_BDC MOF/1, 2, 5 wt% reduced graphitic carbon hybrids.

Raman spectrum obtained after interaction and scattering of electromagnetic radiation with matter gives an idea about crystalline and the defects rich nature of material^63–67^. Raman spectra of NiNH_2_BDC MOF rGO hybrids have been presented in Fig. 5. In the selected range of analysis (4000–500 cm^−1^) the G and D band appears due to graphitic carbon vibrations and carbon defects, respectively. The calculated I_D_ /I_G_ value is in following order; NiNH_2_BDC (0.95) < 1 wt% rGO composite (0.98) < 2 wt% rGO composite (1.003) < 5 wt% rGO composite (1.008). The (a) amplified I_D_/I_G_ ratio in 5 wt% rGO composite due to rearrangements and structural defects promote the extensive π bonding and electron transfer from donor towards acceptor sites, and (b) ~ 5 and ~ 65-time shifting of G and D bands results in the smooth charge transfer process. Both of these factors are attributed to the enhanced catalytic activity of a 5 wt% reduced graphitic carbon (rGO) hybrid than parent MOF^68^.

**Figure 6:**
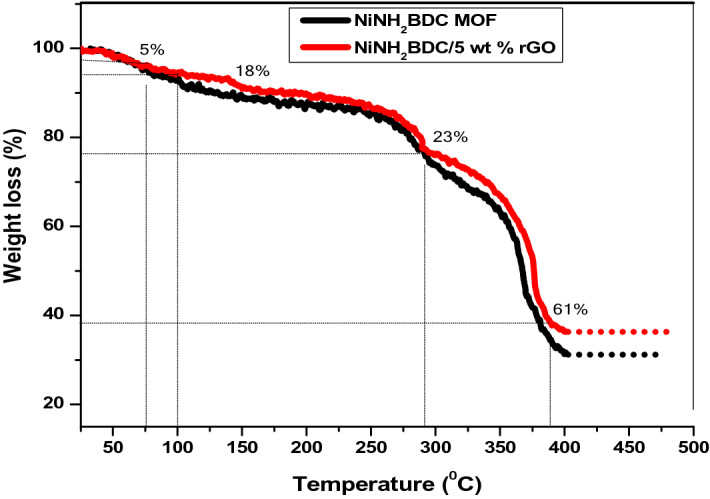
The Thermogramms of NiNH_2_BDC MOF and NiNH_2_BDC/5 wt% reduced graphitic carbon hybrids.

The TGA (Thermo gravimetric analysis) was performed for the investigation of the stability of the electrocatalysts. The thermal behavior of MOF and 5 wt% rGO composite is divided into four domains of mass loss (Fig. 6). The first step at 75 °C corresponded to the solvated DMF molecules, (5% loss). A second step between 100–240 °C was assigned to surface adsorbed water loss (18%), The third step at 290 °C corresponds to the loss of coordinated DMF(23% loss), and after that 61% weight loss at 388 °C represents structure collapse with the linker decomposition (Fig. 6)^69–72^.

### Electrochemical testing of NiNH_2_BDC MOF/1, 2, 5 wt% reduced graphitic carbon hybrids for MOR

**Figure 7:**
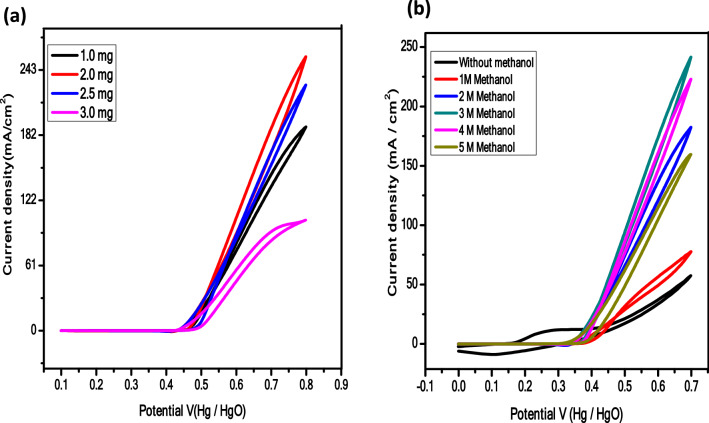
(**a**) Influence of electrocatalyst deposited quantity, and (**b**) CH_3_OH molarity on the current density response of NiNH_2_BDC MOF/1, 2, 5 wt% reduced graphitic carbon hybrids during the optimization process.

The electrocatalyst (1–3 mg) was mounted on GCE to evaluate the optimized catalyst amount. The gradual increase in current density by increasing catalyst amount was due to easy access to abundant and exposed electroactive sites and after that decline in current density is due to; (i) restricted utilization of bottom layered material due to upper thick layer (ii) inferior charge transfer process, and (iii) active sites blockage by reaction intermediates (Fig. 7a)^73–76^. Likewise, during methanol concentration optimization (1–5 M), the 3 M concentration with the maximum delivered current was found to be the optimum amount. At low content of CH_3_OH, the boosted current response is owing to excess of available OH^−^ ions owing to diffusion-controlled methanol transport process while at high CH_3_OH concentration, excess of methanol limit the OH^−^ adsorption, and reaction intermediates block the active sites and consequently depress the catalytic activity, Fig. 7b^77–79^.

**Figure 8:**
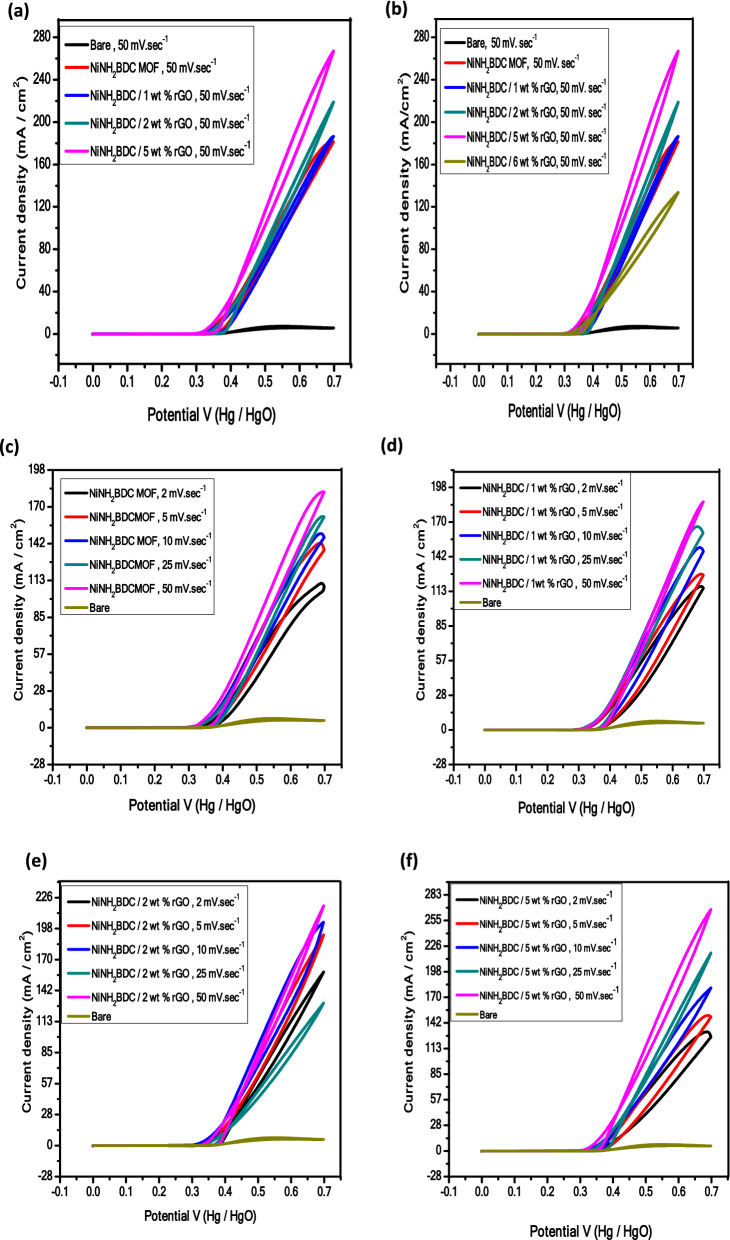
Cyclic Voltamogram of (**a**) NiNH_2_BDC MOF/1–5 wt% reduced graphitic carbon hybrids at 50 mV/s (**b**) NiNH_2_BDC MOF/1–6 wt% reduced graphitic carbon hybrids at 50 mV/s (**c**) NiNH_2_BDC MOF (**d**) NiNH_2_BDC/1 wt% reduced graphitic carbon hybrids (**e**) NiNH_2_BDC/2 wt% reduced graphitic carbon hybrids, and (**f**) NiNH_2_BDC/5 wt% reduced graphitic carbon hybrids in 3 M CH_3_OH/1 M NaOH solution at scanning speed 2–50 mV/s.

The electrocatalytic response of all samples in the presence and absence of CH_3_OH is compared and analyzed at 50 mV/s in Fig. 8a,b. Without methanol, the 5.76 mA/cm^2^ current density is produced while current density response of NiNH_2_BDC MOF/1–5 wt% reduced graphitic carbon hybrids is 181.01 (NiNH_2_BDC) < 186.86 (1 wt% rGO) < 218.94 ( 2 wt% rGO) < ,and 267.77 (5 wt% rGO). Among all the analyzed composites the prime reason for the extraordinary current density of NiNH_2_BDC/5 wt% rGO composite is; (a) synergistic effect between MOF and rGO (b) enhanced surface area, greater stability, and excellent conductivity due to sheet-like morphology of rGO. However, the excessive rGO amount in NiNH_2_BDC/6 wt% rGO composite results in (i) rGO sheets restacking due to pi–pi interaction and choked active sites (ii) inhibited methanol diffusion, and (iii) firmly attached reaction intermediate (CO) limiting OH^−^ ions adsorption on electrode surface^24,53,80–82^.

The Cyclic Voltametric investigations were executed at the scanning speed of 2, 5, 10, 25, and 50 mV/s by selecting the voltage window of − 0.1 to 0.7 to recognize the influence of scanning speed on the current density of the tested samples. At the highest scanning speed, the easy and maximum approach of electroactive species towards the electrode surface leads to maximum current density Fig. 8b–f^83,84^.

**Figure 9:**
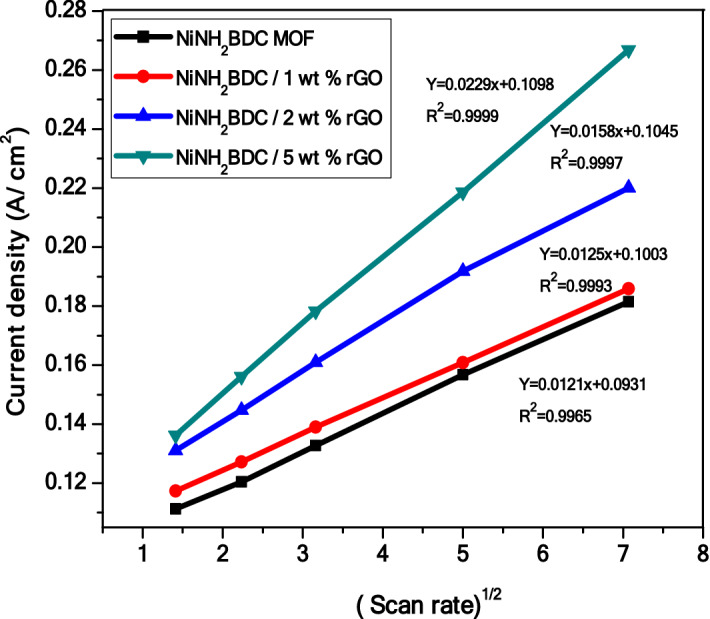
A graphical representation of the direct relationship of peak current density (j) versus under root of scan rate (ν) in 3 M CH_3_OH/1 M NaOH solution.

To get information about the diffusion-controlled process, a straight line obtained by directly relating the peak current density with (scan rate)^1/2^, provides a slop that is equivalent to diffusion coefficient (Fig. 9). Furthermore, the diffusion coefficient (D) is calculated by inserting the value of the α (charge transfer coefficient) in the Randles Sevcik equation ^85^.

$${\text{Ip}} = \left( {2.99 \times 10^{5} } \right){\text{n}}\left( {\upalpha \;{\text{n}}_{\upalpha } } \right)^{{1/2}} {\text{A}}\;{\text{C}}\;{\text{D}}^{{1/2}} v^{{1/2}}$$

For non-reversible oxidation process, the calculated D (diffusion coefficient) value are; 13.2 × 10^−5^ cm^2^ s^−1^ for NiNH_2_BDC MOF, 16.1 × 10^−5^ cm^2^ s^−1^ for 1 wt% rGO composite, 21.8 × 10^−5^ cm^2^ s^−1^ for 2wt% rGO composite, and 31.8 × 10^−5^ cm^2^ s^−1^ for 5wt% rGO composite, respectively (Table 1). R^2^ value ≈ 1 and absolute value of diffusion co-efficient for NiNH_2_BDC 5 wt% rGO composite prove it to be active MOR catalyst^36^.

**Table 1:** The comparative statement of the magnitude of the diffusion coefficient and R^2^ of NiNH_2_BDC MOF/1, 2, 5 wt% reduced graphitic carbon hybrids at 50 mV/s.

Electrocatalyst	R^2^	Diffusion coefficient (cm^2^ s^−1^)
NiNH_2_BDC MOF	0.9991	13.2 × 10^−5^
NiNH_2_BDC/1 wt% rGO	0.9993	16.1 × 10^−5^
NiNH_2_BDC/2 wt% rGO	0.9996	21.8 × 10^−5^
NiNH_2_BDC/5 wt% rGO	0.9997	31.8 × 10^−5^

**Figure 10:**
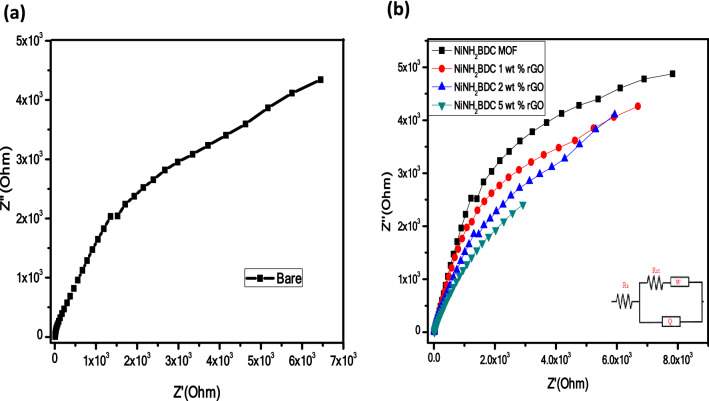
Nyquist plot (**a**) Bare and (**b**) NiNH_2_BDC MOF/1, 2, 5 wt% reduced graphitic carbon hybrids in 3 M CH_3_OH/1 M NaOH solution at oxidation potential 0.699 V.

The EIS (Electrochemical Impedance Spectroscopy), an important parameter tends to explore the kinetics and reaction mechanism. The EIS evaluation was accomplished in 3 electrode systems within the selected frequency domain of 1–1 × 10^5^ Hz in an alkaline solution. Figure 10a,b illustrate the Nyquist plot of the bare electrode and NiNH_2_BDC MOF/rGO composites. A depressed semicircle of NiNH_2_BDC/5 wt% rGO hybrid illustrates the lowermost resistance (highest conductivity) than other counterparts due to homogenous scattering of MOF NPs on rGO surface having high surface area, exposed electroactive sites with maximum OH^−^ adsorption, facile CO oxidation, smooth charge, and mass transfer, and accelerated CH_3_OH oxidation process^86^. Besides, modification of interfacial structure as a result of rGO inclusion is also an important kinetic controlling factor^87–89^. The extracted EIS data obtained after fitting a suitable circuit represent the minimum contact resistance, electrolyte resistance, and substrate inherent resistance with consequent excellent conductivity and electrocatalytic activity of NiNH_2_BDC/5 wt% rGO (Table 2)^90,91^.

**Table 2:** CPE (Q), Warburg diffusion coefficient (W), charge transfer resistance (Rct), and resistance of solution (Rs) of NiNH_2_BDC MOF/1, 2, 5 wt% reduced graphitic carbon hybrids plucked from EIS information of MOR process.

Electrocatalyst	Rs (Ω)	Rct (Ω)	Q (S^n^ Ω^−1^)	W (Ω)
Bare	18.84	86.24	492.1 × 10^−4^	33.89 × 10^−6^
NiNH_2_BDC MOF	14.24	67.13	538.5 × 10^−4^	39.97 × 10^−6^
NiNH_2_BDC/1 wt% rGO	14.10	63.10	540.8 × 10^−4^	40.09 × 10^−6^
NiNH_2_BDC/2 wt% rGO	13.33	58.35	548.8 × 10^−4^	43.16 × 10^−6^
NiNH_2_BDC/5 wt% rGO	13.26	35.50	1.072 × 10^−2^	92.33 × 10^−6^

**Figure 11:**
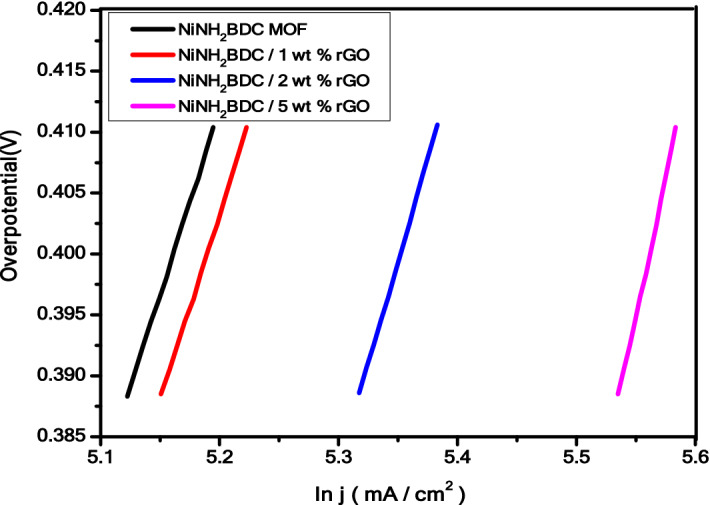
Tafel plot (η vs ln j) of NiNH_2_BDC MOF/1, 2, 5 wt% reduced graphitic carbon hybrids in 3 M CH_3_OH/1 M NaOH solution.

Tafel plot is another important parameter utilized for evaluation of methanol oxidation activity, reaction mechanism, and kinetics of catalytic process by co-relating ln current density (ln j) with overpotential (η) (Fig. 11). Over potential is the required potential greater than the requisite potential for a reaction to occur. The kinetic behavior of as-synthesized samples is determined by the given below Tafel equation ^92^.

$$\upeta = {\text{A}} + {\text{b}}\;\log \;{\text{j}}$$

At low overpotential (0.388 V), the NiNH_2_BDC MOF/1, 2, 5 wt% rGO composites calculated Tafel slope values are in order of; 62.0 (pure MOF) > 61.6 (1 wt% rGO) > 59.6 (2 wt% rGO) > 57.3 mV/dec (5 wt% rGO), respectively. Moreover,at comparatively greater overpotential (0.410 V), the Tafel slope follow the sequence of; 65.4 (pure MOF) > 65.1(1 wt% rGO) > 63.0 (2 wt% rGO) > 60.8 mV/dec (5 wt% rGO) (Table 3). Two different informations are obtained by calculating the Tafel slope at low and high overpotential region as (a) Hydrogen removal (dehydrogenation) from CH_3_OH is the rate controlling step in low potential region while (b) CO exclusion during oxidation process occur in the escalated potential domain. The NiNH_2_BDC/5 wt% rGO composite lowermost Tafel slope value (57.3 mV/dec) reflects the fast removal of hydrogen from CH_3_OH during oxidation process^93–97^.

**Table 3:** The Tafel slope values of NiNH_2_BDC MOF/1, 2, 5 wt% reduced graphitic carbon hybrids at high and low overpotential.

Electrocatalyst	Tafel slope (mV dec^−1^) η = 0.388 V	Tafel slope (mV dec^−1^) η = 0.410 V
NiNH_2_BDC MOF	62.0	65.4
NiNH_2_BDC/1 wt% rGO	61.6	65.1
NiNH_2_BDC/2 wt% rGO	59.6	63.1
NiNH_2_BDC/5 wt% rGO	57.3	60.8

**Figure 12:**
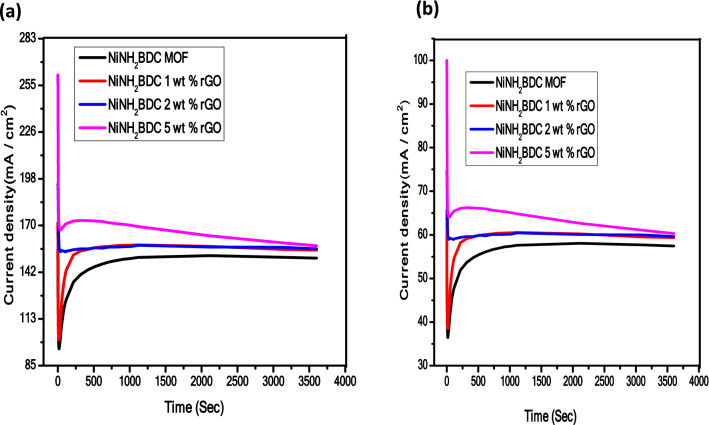
(**a**) The stability trend of NiNH2BDC MOF/1, 2, 5 wt% reduced graphitic carbon hybrids and (**b**) % stability retained by all samples in 3 M CH_3_OH/1 M NaOH solution at oxidation potential of 0.69 V vs Hg/HgO for 3600 s.

The utmost requirement for the practical application of electrocatalyst is its long-term stability under experimental conditions. A Chronoamperometry (*i*/*t*) experiment is conducted in N_2_ saturated alkali solution at peak potential 0.69 V vs Hg/HgO in three electrodes set up for 60 min. The graphical response of the oxidation process can be elaborated as (i) initial maximum j (current density) is due to strong binding of catalyst at the electrode surface, minimum gas bubbles, and large available active sites. (ii) The rapid decline in current density after a short period is associated with (a) extreme gas release and reaction intermediates formation which block the electroactive sites, and(b) material detachment due to excessive bubbling (iii) finally, a steady-state is achieved which persists for 3600 s due to the reaction intermediates passive adsorption^98–101^. According to chronoamperometry graph, the stability retained by NiNH_2_BDC/5 wt% rGO composite is 60.6% while the stability retained by remaining samples is; NiNH_2_BDC/2 wt% rGO 59.3%, NiNH_2_BDC/1 wt% rGO 59.0%, and NiNH_2_BDC MOF 57.3%, respectively (Fig. 12a,b). The minimum loss in current density of NiNH_2_BDC/5 wt% rGO composite attributed to (a) large specific surface area provided by 2D rGO sheets (b) tolerance towards poisonous reaction intermediates (c) fine scattering of small size MOF nanoparticles on rGO surface, and trivial charge transfer resistance^96^.

**Figure 13:**
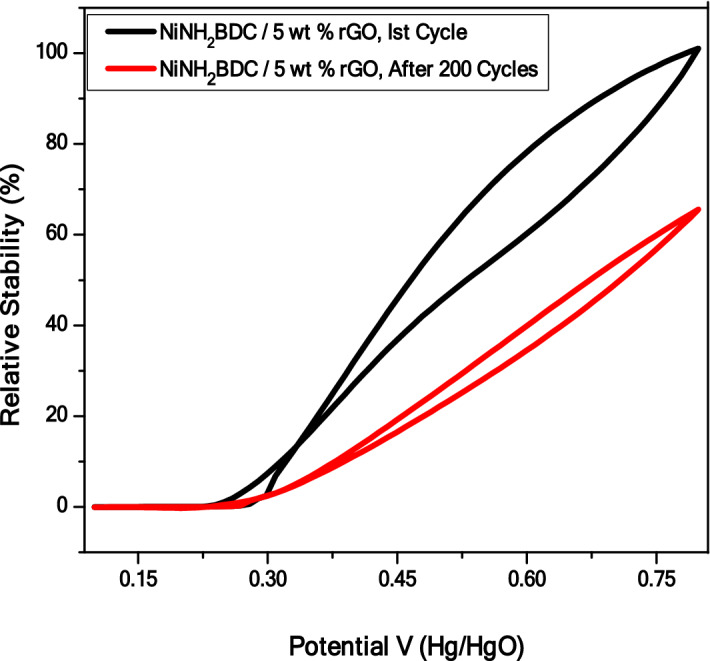
(**a**) The Cyclic stability test of NiNH2BDC MOF/5 wt% reduced graphitic carbon hybrids in 3 M CH_3_OH/1 M NaOH solution at 50 mV/s.

The greater stability of NiNH_2_BDC/5 wt% rGO composite was further evaluated through Cyclic Voltamogram. The current density reserved by electrocatalyst after 200 cycles are presented in Fig. 13. The sample stability tends to decline after successive cycling due to the blockage of active sites owing to excessive bubbling during MOR with electrode surface coverage, inhibited transport of electrolyte toward electrocatalytic material, and decrease in Electrochemical Active Surface Area. This problem can be settled by refreshing the electrolyte via a subsequent cathodic reduction in reverse scan and by performing the CV for few cycles^98,102,103^.

The recommended mechanism for the CH_3_OH oxidation process is as under^104,105^.

1 $${\text{Ni}}^{0} + 2{\text{OH}}^{ - } \to {\text{Ni}}^{{ + 2}} \left( {{\text{OH}}} \right)_{2}$$

2 $${\text{Ni}}^{{ + 2}} \left( {{\text{OH}}} \right)_{2} \to {\text{Ni}}^{{ + 3}} \left( {{\text{OH}}} \right)_{2} + {\text{e}}^{ - }$$

3 $${\text{Ni}}^{{ + 3}} \left( {{\text{OH}}} \right)_{2} + {\text{OH}}^{ - } \to {\text{Ni}}^{{ + 3}} \left( {{\text{OH}}} \right)_{3}$$

4 $${\text{Ni}}^{{ + 3}} \left( {{\text{OH}}} \right)_{3} \to {\text{NiOOH}} + {\text{H}}_{2} {\text{O}}$$

5 $${\text{NiOOH}} + {\text{CH}}_{3} {\text{OH}}_{{({\text{ads}})}} \to {\text{Ni}}^{{ + 2}} \left( {{\text{OH}}} \right)_{2} + \left( {{\text{CH}}_{2} {\text{O}}} \right)_{{{\text{ads}}}}$$

6 $${\text{NiOOH}} + \left( {{\text{CH}}_{2} {\text{O}}} \right)_{{({\text{ads}})}} \to {\text{Ni}}^{{ + 2}} \left( {{\text{OH}}} \right)_{2} + \left( {{\text{CHO}}} \right)_{{{\text{ads}}}}$$

7 $${\text{NiOOH}} + \left( {{\text{CHO}}} \right)_{{({\text{ads}})}} \to {\text{Ni}}^{{ + 2}} \left( {{\text{OH}}} \right)_{2} + \left( {{\text{CO}}} \right)_{{{\text{ads}}}}$$

8 $${\text{NiOOH}} + \left( {{\text{CHO}}} \right)_{{({\text{ads}})}} \to {\text{Ni}}^{{ + 2}} \left( {{\text{OH}}} \right)_{2} + {\text{CO}}_{2}$$

In the case of a Nickel-based system, NiO smoothened the CO oxidation by providing required oxygen while the NiOOH group promotes the MOR by Ni^+2^/Ni^+3^ oxidation/reduction process where + 2 to + 3 oxidation further promote CO oxidation^35,106^.

The mass activity of all electrocatalysts is determined from the ratio of the current density vs deposited mass

$${\text{Mass}}\;{\text{Activity}} = {\text{J}}/{\text{m}}$$

The Mass Activity (M.A) of tested samples at an overpotential of 0.331 V is as under; NiNH_2_BDC MOF 113.8 mA/mg < NiNH_2_BDC/1 wt% rGO composite 120.4 mA/mg < NiNH_2_BDC/2 wt% rGO composite 133.8 mA/mg < and NiNH_2_BDC/5 wt% rGO composite 168.7 mA/mg.

To calculate the electrocatalytic activity of as-synthesized samples, EASA is determined by dividing Cdl (double-layer capacitance) with Cs (specific capacitance). The specific capacitance is a constant factor for each specific system while double-layer capacitance is determined through multiple CV scans or EIS in the non-faradic region. The estimated EASA of NiNH_2_BDC MOF/1, 2, 5 wt% reduced graphitic carbon hybrids at voltage 0.19 was observed to be; 7.6 (pure MOF) < 9.1 (1 wt% rGO) < 14.4 (2 wt% rGO) < 15.7(5 wt% rGO), correspondingly. The comparatively heightened catalytically active surface area of NiNH_2_BDC/5 wt% rGO composite proves the superb electrocatalytic performance of material for MOR (Supplementary information Fig. 1). The given data also authenticate the CV, EIS, and Tafel results.

Moreover, the EASA is divided by the geometrical area of the electrode to calculate the R.F (roughness factor). It is a unitless factor, as it is a ratio.

$${\text{R}}.{\text{F}} = {\text{~EASA}}/{\text{Electrode}}\;{\text{geometrical}}\;{\text{area}}$$

**Figure 14:**
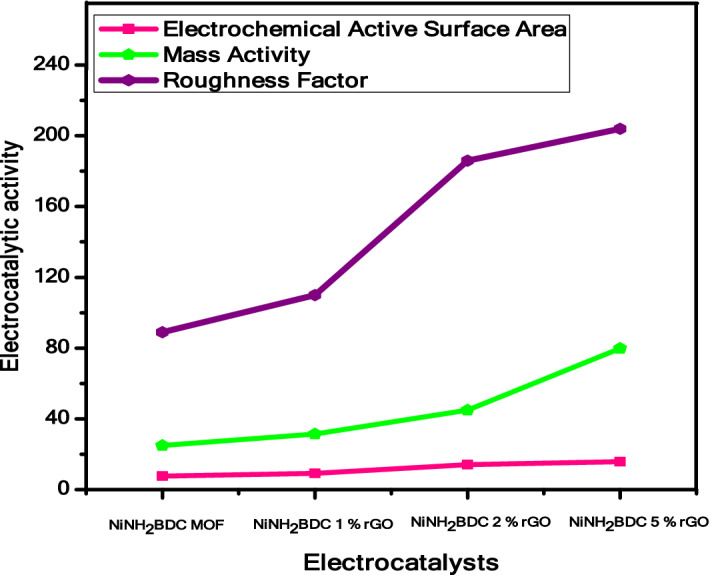
The comparison of EASA, Mass activity, and Roughness factor of NiNH_2_BDC MOF/1, 2, 5 wt% reduced graphitic carbon hybrids.

The heightened roughness factor reflects the excellent catalytic performance of material due to the direct relationship between EASA and R.F. Roughness factor of NiNH_2_BDC MOF/1–5 wt% reduced graphitic carbon hybrids were found in the following order; 107, 128, 204, and 222 respectively (Table 4 and Fig. 14)^107^.

**Table 4:** EASA, Mass activity, and Roughness factor comparison of NiNH_2_BDC MOF/1, 2, 5 wt% reduced graphitic carbon hybrids.

Catalyst	EASA (cm^2^)	Mass activity (mA/mg)	Roughness factor
NiNH_2_BDC MOF	7.6	113.8	107
NiNH_2_BDC/1 wt% rGO	9.1	120.3	128
NiNH_2_BDC/2 wt% rGO	14.1	133.8	204
NiNH_2_BDC/5 wt% rGO	15.7	168.7	222

The comparative statement of the electrocatalytic response of tested materials with already reported materials is provided in Table 5 given below.

**Table 5:** The electrocatalytic activity of synthesized samples in comparison with reported materials.

Electrocatalytic materials	Molarity of Methanol solution (M)	Scan rate (mV s^−1^)	Oxidation potential (V) versus RHE	Anodic current density (mA cm^−2^)	Resistance (Ω)	References
Pt/rGO	3	50	1.72	32	–	^108^
NiO-MOF/rGO	3	50	1.83	276	22.8	^23^
Cu BTC/5 wt% rGO	3	50	1.84	120	20.53	^22^
Ni Cr LDH	3	50	1.63 V	7.02	–	^109^
NiNH_2_BDC MOF	3	50	1.614 V	180.0	14.24	This work
NiNH_2_BDC/1 wt% rGO	3	50	1.614 V	186.8	14.10	This work
NiNH_2_BDC/2 wt% rGO	3	50	1.614 V	218.94	13.33	This work
NiNH_2_BDC/5 wt% rGO	3	50	1.614 V	267.77	13.26	This work

## Conclusions

The NiNH_2_BDC MOF/1–5 wt% reduced graphitic carbon hybrids (NiNH_2_BDC/rGO) fabricated by sonication-assisted solvothermal approach were studied for the CH_3_OH oxidation process under alkaline condition. The NiNH_2_BDC MOF/5 wt% rGO composite by possessing auspicious current of 267.7 mA cm^−2^ at voltage 0.69, Tafel slope of 60.8 mV dec^−1^, the resistance of 13.26 Ω, EASA 15.7 cm^2^, mass activity 168.7 mA/mg and roughness factor 222 in 3 M CH_3_OH/1 M NaOH solution displays better activity as compared to the state-of-the-art platinum-based materials and prove to be a proficient substitute of costly materials exploited for MOR in the direct CH_3_OH fuel cell.

## Supplementary Information

Supplementary Information.

## References

[1] Chu S, Majumdar A (2012). Opportunities and challenges for a sustainable energy future. Nature.

[2] Reddy ALM, Gowda SR, Shaijumon MM, Ajayan PM (2012). Hybrid nanostructures for energy storage applications. Adv. Mater..

[3] Wang H, Dai H (2013). Strongly coupled inorganic–nano-carbon hybrid materials for energy storage. Chem. Soc. Rev..

[4] Choi NS, Chen Z, Freunberger SA, Ji X, Sun YK, Amine K (2012). Challenges facing lithium batteries and electrical double-layer capacitors. Angew. Chem. Int. Ed..

[5] Steele, B. C. & Heinzel, A. Materials for fuel-cell technologies. In *Materials for Sustainable Energy: A Collection of Peer-Reviewed Research and Review Articles from Nature Publishing Group*, 224–231 (World Scientific, 2011).

[6] Coutanceau C, Koffi R, Léger J-M, Marestin K, Mercier R, Nayoze C (2006). Development of materials for mini DMFC working at room temperature for portable applications. J. Power Sources.

[7] Borello D, Calabriso A, Cedola L, Del Zotto L, Santori SG (2014). Development of improved passive configurations of DMFC with reduced contact resistance. Energy Procedia.

[8] Wang L, Yuan Z, Wen F, Cheng Y, Zhang Y, Wang G (2018). A bipolar passive DMFC stack for portable applications. Energy.

[9] Dillon R, Srinivasan S, Arico A, Antonucci V (2004). International activities in DMFC R&D: status of technologies and potential applications. J. Power Sources.

[10] Shrivastava NK, Thombre SB, Chadge RB (2016). Liquid feed passive direct methanol fuel cell: challenges and recent advances. Ionics.

[11] Chen R, Zhao T (2005). Mathematical modeling of a passive-feed DMFC with heat transfer effect. J. Power Sources.

[12] Gwak G, Lee K, Ferekh S, Lee S, Ju H (2015). Analyzing the effects of fluctuating methanol feed concentration in active-type direct methanol fuel cell (DMFC) systems. Int. J. Hydrogen Energy.

[13] Zhao T, Chen R, Yang W, Xu C (2009). Small direct methanol fuel cells with passive supply of reactants. J. Power Sources.

[14] Hanif S, Iqbal N, Shi X, Noor T, Ali G, Kannan A (2020). NiCo-N-doped carbon nanotubes based cathode catalyst for alkaline membrane fuel cell. Renew. Energy.

[15] Rizvi SAM, Iqbal N, Haider MD, Noor T, Anwar R, Hanif S (2019). Synthesis and Characterization of Cu-MOF Derived Cu@ AC Electrocatalyst for Oxygen Reduction Reaction in PEMFC. Catal. Lett..

[16] Hu H, Cheng H, Zhou J, Zhu Q, Yu Y (2017). Hierarchical porous Fe_2_O_3_ assisted with graphene-like carbon as high-performance lithium battery anodes. Mater. Today Phys..

[17] Hacquard, A. Improving and understanding direct methanol fuel cell (DMFC) performance (WORCESTER POLYTECHNIC INSTITUTE, 2005).

[18] Yaqoob L, Noor T, Iqbal N (2021). Recent progress in development of efficient electrocatalyst for methanol oxidation reaction in direct methanol fuel cell. Int. J. Energy Res..

[19] Reddington E, Sapienza A, Gurau B, Viswanathan R, Sarangapani S, Smotkin ES (1998). Combinatorial electrochemistry: a highly parallel, optical screening method for discovery of better electrocatalysts. Science.

[20] Lin Y, Cui X, Yen CH, Wai CM (2005). PtRu/carbon nanotube nanocomposite synthesized in supercritical fluid: a novel electrocatalyst for direct methanol fuel cells. Langmuir.

[21] Cao L, Scheiba F, Roth C, Schweiger F, Cremers C, Stimming U (2006). Novel nanocomposite Pt/RuO_2_·x H_2_O/carbon nanotube catalysts for direct methanol fuel cells. Angew. Chem. Int. Ed..

[22] Noor T, Ammad M, Zaman N, Iqbal N, Yaqoob L, Nasir H (2019). A highly efficient and stable copper BTC metal organic framework derived electrocatalyst for oxidation of methanol in DMFC application. Catal. Lett..

[23] Noor T, Zaman N, Nasir H, Iqbal N, Hussain Z (2019). Electro catalytic study of NiO-MOF/rGO composites for methanol oxidation reaction. Electrochim. Acta.

[24] Bai L (2018). Synthesis of PtRu/Ru heterostructure for efficient methanol electrooxidation: the role of extra Ru. Appl. Surf. Sci..

[25] Watanabe M, Motoo S (1975). Electrocatalysis by ad-atoms: part II. Enhancement of the oxidation of methanol on platinum by ruthenium ad-atoms. J. Electroanal. Chem. Interfacial Electrochem..

[26] Du W, Bai Y-L, Xu J, Zhao H, Zhang L, Li X (2018). Advanced metal-organic frameworks (MOFs) and their derived electrode materials for supercapacitors. J. Power Sources.

[27] Zhang T, Jin Y, Shi Y, Li M, Li J, Duan C (2019). Modulating photoelectronic performance of metal–organic frameworks for premium photocatalysis. Coord. Chem. Rev..

[28] Toyao T, Saito M, Horiuchi Y, Mochizuki K, Iwata M, Higashimura H (2013). Efficient hydrogen production and photocatalytic reduction of nitrobenzene over a visible-light-responsive metal–organic framework photocatalyst. Catal. Sci. Technol..

[29] Hickson T (2011). Identifying the top 20 per cent. Interview by Christian Martin. Nat. Mater..

[30] Hassan MH, Soliman AB, Elmehelmey WA, Abugable AA, Karakalos SG, Elbahri M (2019). A Ni-loaded, metal–organic framework–graphene composite as a precursor for in situ electrochemical deposition of a highly active and durable water oxidation nanocatalyst. Chem. Commun..

[31] Sarwar E, Noor T, Iqbal N, Mehmood Y, Ahmed S, Mehek R (2018). Effect of Co–Ni ratio in graphene based bimetallic electro-catalyst for methanol oxidation. Fuel Cells.

[32] Li X, Lei H, Yang C, Zhang Q (2018). Electrochemical fabrication of ultra-low loading Pt decorated porous nickel frameworks as efficient catalysts for methanol electrooxidation in alkaline medium. J. Power Sources.

[33] Guerrero-Ortega L, Manzo-Robledo A, Ramírez-Meneses E, Mateos-Santiago J, Lartundo-Rojas L, Garibay-Febles V (2018). Methanol electro-oxidation reaction at the interface of (bi)-metallic (PtNi) synthesized nanoparticles supported on carbon Vulcan. Int. J. Hydrogen Energy.

[34] Ferrer DM, Banda JAM, Rodrigo RS, Gómez JYV, García UP, Vicente PDÁ (2018). Electrochemical performance of Pt/NC and Pt/rGO for methanol oxidation in acid media. ECS Trans..

[35] Raoof JB, Ojani R, Hosseini SR (2013). An electrochemical investigation of methanol oxidation on nickel hydroxide nanoparticles. S. Afr. J. Chem..

[36] Shahid MM, Pandikumar A, Golsheikh AM, Huang NM, Lim HN (2014). Enhanced electrocatalytic performance of cobalt oxide nanocubes incorporating reduced graphene oxide as a modified platinum electrode for methanol oxidation. RSC Adv..

[37] Candelaria SL, Bedford NM, Woehl TJ, Rentz NS, Showalter AR, Pylypenko S (2017). Multi-component Fe–Ni hydroxide nanocatalyst for oxygen evolution and methanol oxidation reactions under alkaline conditions. ACS Catal..

[38] Li J, Luo Z, Zuo Y, Liu J, Zhang T, Tang P (2018). NiSn bimetallic nanoparticles as stable electrocatalysts for methanol oxidation reaction. Appl. Catal. B.

[39] Hussain S, Ullah N, Zhang Y, Shaheen A, Javed MS, Lin L (2019). One-step synthesis of unique catalyst Ni9S8@ C for excellent MOR performances. Int. J. Hydrogen Energy.

[40] Yaqoob L, Noor T, Iqbal N, Nasir H, Zaman N (2019). Development of nickel-BTC-MOF-derived nanocomposites with rGO towards electrocatalytic oxidation of methanol and its product analysis. Catalysts.

[41] Wang L, Wu Y, Cao R, Ren L, Chen M, Feng X (2016). Fe/Ni metal–organic frameworks and their binder-free thin films for efficient oxygen evolution with low overpotential. ACS Appl. Mater. Interfaces..

[42] Iqbal B, Saleem M, Arshad SN, Rashid J, Hussain N, Zaheer M (2019). One-pot synthesis of heterobimetallic metal-organic frameworks (MOF) for multifunctional catalysis. Chem. Eur. J..

[43] Wu Z-S, Ren W, Gao L, Liu B, Jiang C, Cheng H-M (2009). Synthesis of high-quality graphene with a pre-determined number of layers. Carbon.

[44] Cao, N. & Zhang, Y. Study of reduced graphene oxide preparation by Hummers’ method and related characterization. *J. Nanomat.* 168125, (2015).

[45] Wu B, Lin X, Ge L, Wu L, Xu T (2013). A novel route for preparing highly proton conductive membrane materials with metal-organic frameworks. Chem. Commun..

[46] Vu TA, Le GH, Dao CD, Dang LQ, Nguyen KT, Dang PT (2014). Isomorphous substitution of Cr by Fe in MIL-101 framework and its application as a novel heterogeneous photo-Fenton catalyst for reactive dye degradation. RSC Adv..

[47] Guo H, Zheng Z, Zhang Y, Lin H, Xu Q (2017). Highly selective detection of Pb^2+^ by a nanoscale Ni-based metal–organic framework fabricated through one-pot hydrothermal reaction. Sens. Actuators, B Chem..

[48] Mesbah A, Rabu P, Sibille R, Lebègue S, Mazet T, Malaman B (2014). From hydrated Ni_3_(OH)_2_(C_8_H_4_O_4_)_2_(H_2_O)_4_ to anhydrous Ni_2_(OH)_2_(C_8_H_4_O_4_): impact of structural transformations on magnetic properties. Inorg. Chem..

[49] Zhang Z, Li X, Liu B, Zhao Q, Chen G (2016). Hexagonal microspindle of NH 2-MIL-101 (Fe) metal–organic frameworks with visible-light-induced photocatalytic activity for the degradation of toluene. RSC Adv..

[50] Arul P, John SA (2018). Size controlled synthesis of Ni-MOF using polyvinylpyrrolidone: new electrode material for the trace level determination of nitrobenzene. J. Electroanal. Chem..

[51] Vuong G-T, Pham M-H, Do T-O (2013). Synthesis and engineering porosity of a mixed metal Fe 2 Ni MIL-88B metal–organic framework. Dalton Trans..

[52] Iwasaki T, Yoshii H, Nakamura H, Watano S (2012). Simple and rapid synthesis of Ni–Fe layered double hydroxide by a new mechanochemical method. Appl. Clay Sci..

[53] Zhou L, Kong X, Gao M, Lian F, Li B, Zhou Z (2014). Hydrothermal fabrication of MnCO3@ rGO composite as an anode material for high-performance lithium ion batteries. Inorg. Chem..

[54] Israr F, Kim DK, Kim Y, Oh SJ, Ng KC, Chun W (2015). Cost effective and low energy consuming hydrothermal synthesis of Ni based MOF. J. Energy Eng..

[55] Wang Z, Dong P, Sun Z, Sun C, Bu H, Han J (2018). Sizes/morphologies tunable NH2-Ni-MOFs electrocatalysts for ultrasensitive C-reactive protein detection via an aptamer binding induced DNA walker-antibody sandwich assay. J. Mater. Chem. B.

[56] Bai Y, Du M, Chang J, Sun J, Gao L (2014). Supercapacitors with high capacitance based on reduced graphene oxide/carbon nanotubes/NiO composite electrodes. J. Mater. Chem. A.

[57] Lian K, Kirk D, Thorpe S (1995). Investigation of a “two-state” tafel phenomenon for the oxygen evolution reaction on an amorphous Ni–Co alloy. J. Electrochem. Soc..

[58] Siang TJ, Bach LG, Singh S, Truong QD, Phuc NHH, Alenazey F (2019). Methane bi-reforming over boron-doped Ni/SBA-15 catalyst: LONGEVITY evaluation. Int. J. Hydrogen Energy.

[59] Wang Z, Dong P, Sun Z, Sun C, Bu H, Han J (2018). NH2-Ni-MOF electrocatalysts with tunable size/morphology for ultrasensitive C-reactive protein detection via an aptamer binding induced DNA walker–antibody sandwich assay. J. Mater. Chem. B.

[60] Fominykh K, Feckl JM, Sicklinger J, Döblinger M, Böcklein S, Ziegler J (2014). Ultrasmall dispersible crystalline nickel oxide nanoparticles as high-performance catalysts for electrochemical water splitting. Adv. Funct. Mater..

[61] Yu X-Y, Feng Y, Guan B, Lou XWD, Paik U (2016). Carbon coated porous nickel phosphides nanoplates for highly efficient oxygen evolution reaction. Energy Environ. Sci..

[62] Wang Z, Dong P, Sun Z, Sun C, Bu H, Han J (2018). NH 2-Ni-MOF electrocatalysts with tunable size/morphology for ultrasensitive C-reactive protein detection via an aptamer binding induced DNA walker–antibody sandwich assay. J. Mater. Chem. B.

[63] Zhang L, Hashimoto Y, Taishi T, Ni Q-Q (2011). Mild hydrothermal treatment to prepare highly dispersed multi-walled carbon nanotubes. Appl. Surf. Sci..

[64] Melvin GJH, Ni Q-Q, Suzuki Y, Natsuki T (2014). Microwave-absorbing properties of silver nanoparticle/carbon nanotube hybrid nanocomposites. J. Mater. Sci..

[65] Santangelo S, Messina G, Faggio G, Lanza M, Milone C (2011). Evaluation of crystalline perfection degree of multi-walled carbon nanotubes: correlations between thermal kinetic analysis and micro-Raman spectroscopy. J. Raman Spectrosc..

[66] Lin Y, Watson KA, Fallbach MJ, Ghose S, Smith JG, Delozier DM (2009). Rapid, solventless, bulk preparation of metal nanoparticle-decorated carbon nanotubes. ACS Nano.

[67] Corio P, Santos A, Santos PS, Temperini MLA, Brar V, Pimenta MA (2004). Characterization of single wall carbon nanotubes filled with silver and with chromium compounds. Chem. Phys. Lett..

[68] Kumaraguru S, Yesuraj J, Mohan S (2020). Reduced graphene oxide-wrapped micro-rod like Ni/Co organic-inorganic hybrid nanocomposite as an electrode material for high-performance supercapacitor. Compos. Part B Eng..

[69] Iqbal B, Saleem M, Arshad SN, Rashid J, Hussain N, Zaheer M (2019). One-pot synthesis of heterobimetallic metal-organic frameworks (MOFs) for multifunctional catalysis. Chem. Eur. J..

[70] Yang Y, Lin R, Ge L, Hou L, Bernhardt P, Rufford TE (2015). Synthesis and characterization of three amino-functionalized metal–organic frameworks based on the 2-aminoterephthalic ligand. Dalton Trans..

[71] Peng MM, Jeon UJ, Ganesh M, Aziz A, Vinodh R, Palanichamy M (2014). Oxidation of ethylbenzene using nickel oxide supported metal organic framework catalyst. Bull. Korean Chem. Soc..

[72] Noor T, Pervaiz S, Iqbal N, Nasir H, Zaman N, Sharif M (2020). Nanocomposites of NiO/CuO Based MOF with rGO: an efficient and robust electrocatalyst for methanol oxidation reaction in DMFC. Nanomaterials.

[73] Xu N, Zhu T, Qiao J, Zhang F, Chen Z (2016). Nitrogen and sulfur co-doped mesoporous carbon as cathode catalyst for H_2_/O_2_ alkaline membrane fuel cell–effect of catalyst/bonding layer loading. Int. J. Hydrogen Energy.

[74] Wu L, Li Q, Wu CH, Zhu H, Mendoza-Garcia A, Shen B (2015). Stable cobalt nanoparticles and their monolayer array as an efficient electrocatalyst for oxygen evolution reaction. J. Am. Chem. Soc..

[75] Fang Y, Li X, Li F, Lin X, Tian M, Long X (2016). Self-assembly of cobalt-centered metal organic framework and multiwalled carbon nanotubes hybrids as a highly active and corrosion-resistant bifunctional oxygen catalyst. J. Power Sources.

[76] Xing M, Kong L-B, Liu M-C, Liu L-Y, Kang L, Luo Y-C (2014). Cobalt vanadate as highly active, stable, noble metal-free oxygen evolution electrocatalyst. J. Mater. Chem. A.

[77] Yu EH, Scott K, Reeve RW, Yang L, Allen RG (2004). Characterisation of platinised Ti mesh electrodes using electrochemical methods: methanol oxidation in sodium hydroxide solutions. Electrochim. Acta.

[78] Fashedemi OO, Ozoemena KI (2013). Enhanced methanol oxidation and oxygen reduction reactions on palladium-decorated FeCo@ Fe/C core–shell nanocatalysts in alkaline medium. Phys. Chem. Chem. Phys..

[79] Yaqoob L, Noor T, Iqbal N, Nasir H, Zaman N, Rasheed L (2020). Development of an efficient non-noble metal based anode electrocatalyst to promote methanol oxidation activity in DMFC. ChemistrySelect.

[80] Zeng G, Chen Y, Chen L, Xiong P, Wei M (2016). Hierarchical cerium oxide derived from metal-organic frameworks for high performance supercapacitor electrodes. Electrochim. Acta.

[81] Hamidipour L, Farzaneh F (2013). Cobalt metal organic framework as an efficient heterogeneous catalyst for the oxidation of alkanes and alkenes. React. Kinet. Mech. Catal..

[82] Parwaiz S, Bhunia K, Das AK, Khan MM, Pradhan D (2017). Cobalt-doped ceria/reduced graphene oxide nanocomposite as an efficient oxygen reduction reaction catalyst and supercapacitor material. J. Phys. Chem. C.

[83] Li Y, Wang G, Ye K, Cheng K, Pan Y, Yan P (2014). Facile preparation of three-dimensional multilayer porous MnO_2_/reduced graphene oxide composite and its supercapacitive performance. J. Power Sources.

[84] Maruthapandian V, Kumaraguru S, Mohan S, Saraswathy V, Muralidharan S (2018). An insight on the electrocatalytic mechanistic study of pristine Ni MOF (BTC) in alkaline medium for enhanced OER and UOR. ChemElectroChem.

[85] Cordeiro C, De Vries M, Cremers T, Westerink B (2016). The role of surface availability in membrane-induced selectivity for amperometric enzyme-based biosensors. Sens. Actuators, B Chem..

[86] Devaraj S, Munichandraiah N (2006). Electrochemical supercapacitor studies of nanostructured α-MnO_2_ synthesized by microemulsion method and the effect of annealing. J. Electrochem. Soc..

[87] Niu L, Li Q, Wei F, Chen X, Wang H (2003). Electrochemical impedance and morphological characterization of platinum-modified polyaniline film electrodes and their electrocatalytic activity for methanol oxidation. J. Electroanal. Chem..

[88] Zhu X, Zhang P, Xu S, Yan X, Xue Q (2014). Free-standing three-dimensional graphene/manganese oxide hybrids as binder-free electrode materials for energy storage applications. ACS Appl. Mater. Interfaces..

[89] Wu Q, Jiang M, Zhang X, Cai J, Lin S (2017). A novel octahedral MnO/RGO composite prepared by thermal decomposition as a noble-metal free electrocatalyst for ORR. J. Mater. Sci..

[90] Yu EH, Scott K, Reeve RW (2003). A study of the anodic oxidation of methanol on Pt in alkaline solutions. J. Electroanal. Chem..

[91] Ye W, Zhang X, Chen Y, Du Y, Zhou F, Wang C (2013). Pulsed electrodeposition of reduced graphene oxide on glass carbon electrode as an effective support of electrodeposited Pt microspherical particles: nucleation studies and the application for methanol electro-oxidation. Int. J. Electrochem. Sci.

[92] Huang T, Mao S, Zhou G, Zhang Z, Wen Z, Huang X (2015). A high-performance catalyst support for methanol oxidation with graphene and vanadium carbonitride. Nanoscale.

[93] Wang W, Li Y, Wang H (2013). Tin oxide nanoparticle-modified commercial PtRu catalyst for methanol oxidation. Micro Nano Lett..

[94] Huang Y, Cai J, Liu M, Guo Y (2012). Fabrication of a novel PtPbBi/C catalyst for ethanol electro-oxidation in alkaline medium. Electrochim. Acta.

[95] Wang H, Da H, Wang R, Ji S (2014). Beef-derived mesoporous carbon as highly efficient support for PtRuIr electrocatalysts and their high activity for CO and methanol oxidation. S. Afr. J. Chem..

[96] Ye W, Zhang X, Chen Y, Du Y, Zhou F, Wang C (2013). Pulsed electrodeposition of reduced graphene oxide on glass carbon electrode as an effective support of electrodeposited Pt microspherical particles: nucleation studies and the application for methanol electro-oxidation. Int. J. Electrochem. Sci.

[97] Wang H, Linkov V, Ji S, Zhang W, Lei Z, Wang R (2012). Highly active, carbon-supported, PdSn nano-core, partially covered with Pt, as catalysts for methanol oxidation. S. Afr. J. Chem..

[98] Mao S, Wen Z, Huang T, Hou Y, Chen J (2014). High-performance bi-functional electrocatalysts of 3D crumpled graphene–cobalt oxide nanohybrids for oxygen reduction and evolution reactions. Energy Environ. Sci..

[99] Huang W, Wang H, Zhou J, Wang J, Duchesne PN, Muir D (2015). Highly active and durable methanol oxidation electrocatalyst based on the synergy of platinum–nickel hydroxide–graphene. Nat. Commun..

[100] Behmenyar G, Akın AN (2014). Investigation of carbon supported Pd–Cu nanoparticles as anode catalysts for direct borohydride fuel cell. J. Power Sources.

[101] Zheng Y, Chen H, Dai Y, Zhang N, Zhao W, Wang S (2015). Preparation and characterization of Pt/TiO_2_ nanofibers catalysts for methanol electro-oxidation. Electrochim. Acta.

[102] Vidales AG, Dam-Quang L, Hong A, Omanovic S (2019). The influence of addition of iridium-oxide to nickel-molybdenum-oxide cathodes on the electrocatalytic activity towards hydrogen evolution in acidic medium and on the cathode deactivation resistance. Electrochim. Acta.

[103] Huang W, Wang H, Zhou J, Wang J, Duchesne PN, Muir D (2015). Highly active and durable methanol oxidation electrocatalyst based on the synergy of platinum–nickel hydroxide–graphene. Nat. Commun..

[104] Jafarian M, Mahjani M, Heli H, Gobal F, Khajehsharifi H, Hamedi M (2003). A study of the electro-catalytic oxidation of methanol on a cobalt hydroxide modified glassy carbon electrode. Electrochim. Acta.

[105] Sun H, Ye Y, Liu J, Tian Z, Cai Y, Li P (2018). Pure Ni nanocrystallines anchored on rGO present ultrahigh electrocatalytic activity and stability in methanol oxidation. Chem. Commun..

[106] Sunitha M, Durgadevi N, Sathish A, Ramachandran T (2018). Performance evaluation of nickel as anode catalyst for DMFC in acidic and alkaline medium. J. Fuel Chem. Technol..

[107] McCrory CC, Jung S, Peters JC, Jaramillo TF (2013). Benchmarking heterogeneous electrocatalysts for the oxygen evolution reaction. J. Am. Chem. Soc..

[108] Li Y, Gao W, Ci L, Wang C, Ajayan PM (2010). Catalytic performance of Pt nanoparticles on reduced graphene oxide for methanol electro-oxidation. Carbon.

[109] Gamil S, El Rouby WM, Antuch M, Zedan I (2019). Nanohybrid layered double hydroxide materials as efficient catalysts for methanol electrooxidation. RSC Adv..

