# Supplementary material for: Electrocatalytic performance of NiNH2BDC MOF based composites with rGO for methanol oxidation reaction
Source: Sci Rep. 2021 Jun 28;11:13402. doi: 10.1038/s41598-021-92660-8 (PMC8238968; doi:10.1038/s41598-021-92660-8)
Supplement: Supplementary file 1 — Supplementary Information. [file 41598_2021_92660_MOESM1_ESM.docx]

**Supplementary information**

1. Diffusion co-efficient calculated by employing *Randles Sevcik* equation

Ip = (2.99 × 10^5^) n (α n_α_) ^1/2^ACD^1/2^*v*^1/2^

Ip = Current in Amperes n_α_ = No of electrons involved in rate determining step

A = Surface area of electrode (0.07065 cm^2^) n = No of electrons transferred in process

D = Diffusion co-efficient (cm^2^/sec) C = Concentration in mole/cm^3^

V = Scan rate in V/sec

- Formula used to calculate α is as under

Ep - Ep / 2 = 1.857RT/ nF α

Ep = Peak potential R = General gas constant (8.3143 J/k/mole)

T = 298 k n = No of electrons transferred in process

F = Farad’s constant (96580) α = Charge transfer co-efficient

1. Formula used to calculate Tafel slope

η = A + b log i

η = Over potential (E-E^0^) A = Surface area of electrode (0.07065)

b = Tafel slope log I = log of current density

1. Value of R^2^ determined by linear fitting
2. Value of Rct, Rs and capacitance determined by circuit selected after EIS.
3. Electrochemical active surface area (EASA)

Double layer capacitance = slope of straight line, obtained by plotting a graph between scan rate and current density. The EASA is then determined by formula given below:

EASA = C_dl_ / C_s_

Where Cs is the specific capacitance of a sample under the specific condition of electrolyte and

CDL is the double layer capacitance in the non-faradic region of voltamogram. For Fe and Ni

System the reported value of *Cs* varies from 0.033 mF to 0.056 mF in alkaline conditions. Hence, we chose an average value (0.040 mF.cm^2^) to get the average value of EASA of our catalyst.

1. Roughness factor (RF)

Roughness factor of each electrocatalyst is further calculated by dividing EASA with geometrical area of electrode (0.07065 cm^2^)

R.F = EASA / Geometrical area

Fig 1 : Graph between scan rate and ∆J at 0.19 V vs Hg/HgO in 1 M NaOH solution

(a)


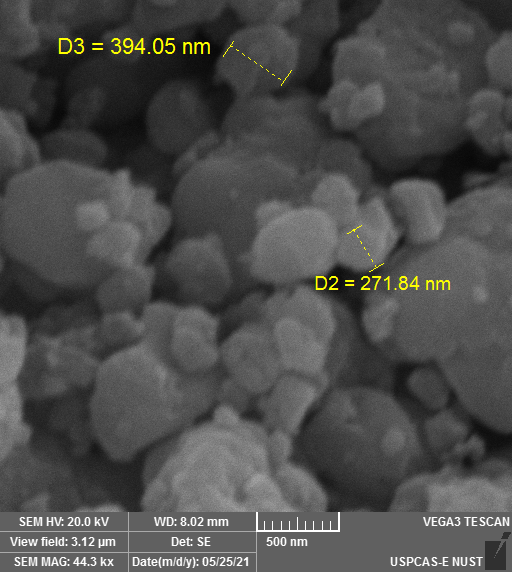

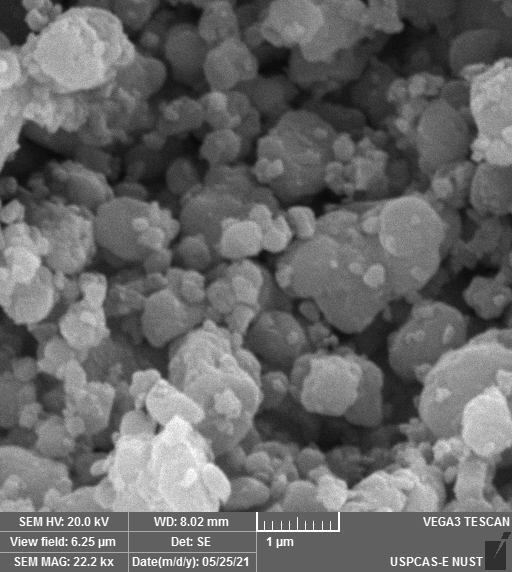


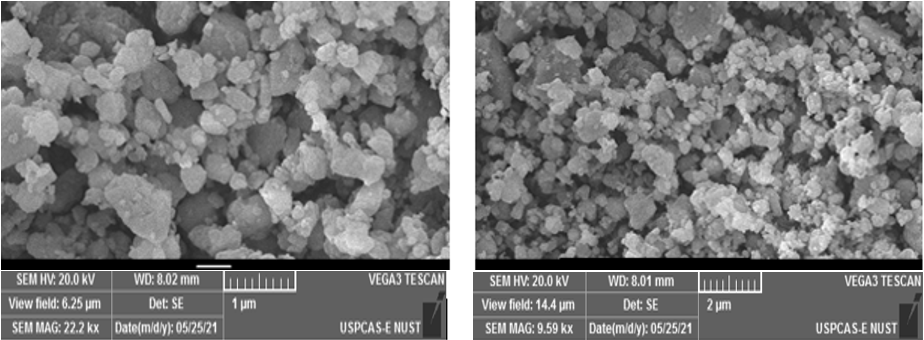


(b)


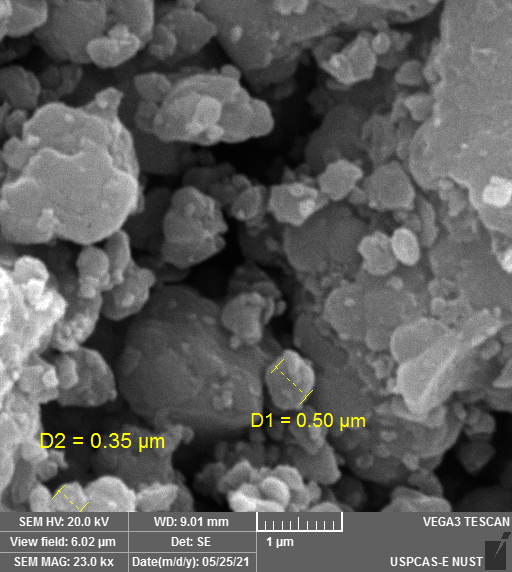

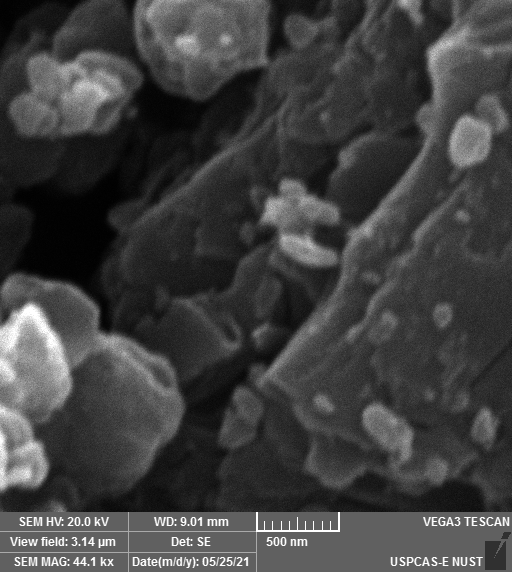


(c)


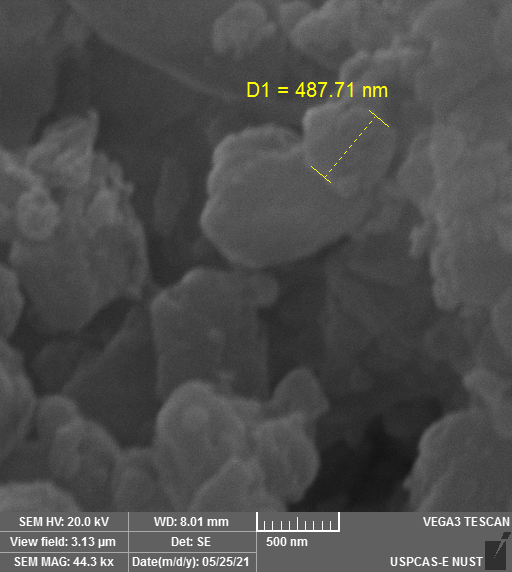

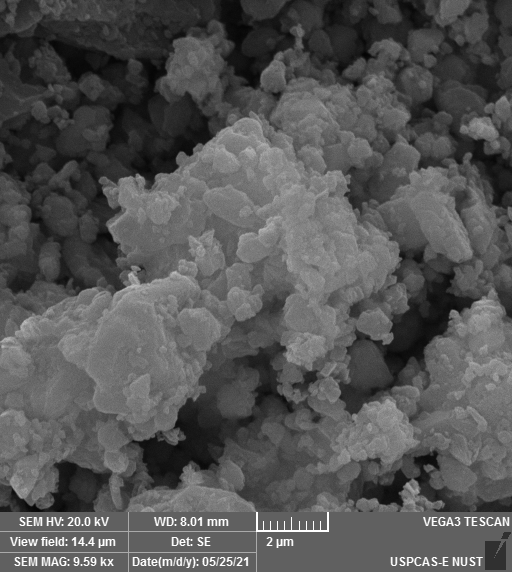


(d)


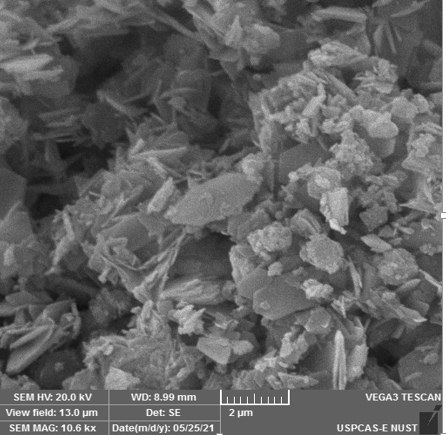

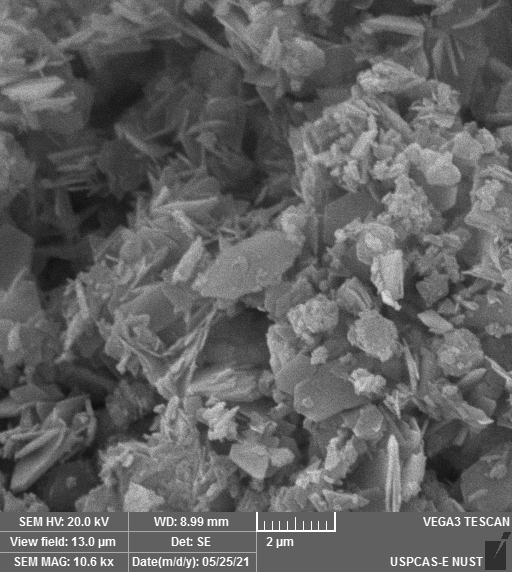


(e)

Figure 2. Scanning electron microscopy images of NiNH_2_BDC MOF (a), 1wt % rGO composite (b), 2 wt % rGO composite (c) 5 wt % rGO composite (d) and Optimization of synthetic conditions (e)at different magnifications.

EDX data

| Electrocatalyst | Wt % of C | Wt % of N | Wt % of O | Wt % of Ni |
| --- | --- | --- | --- | --- |
| NiNH_2_BDC MOF | 34.67 | 9.61 | 42.22 | 13.5 |
| NiNH_2_BDC /1 wt % rGO | 45.34 | 4.19 | 40.65 | 9.83 |
| NiNH_2_BDC /2 wt % rGO | 55.26 | 2.51 | 35.08 | 7.15 |
| NiNH_2_BDC /5 wt % rGO | 65.12 | 0.65 | 30.42 | 3.79 |

EDX Spectra


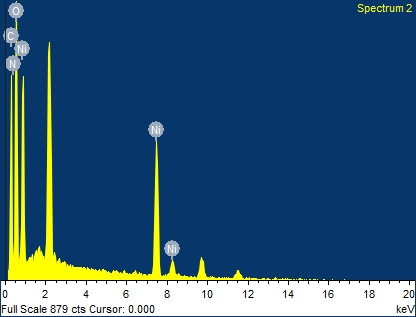

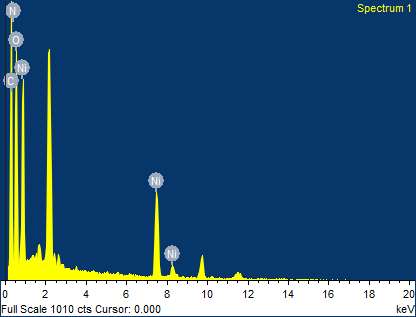


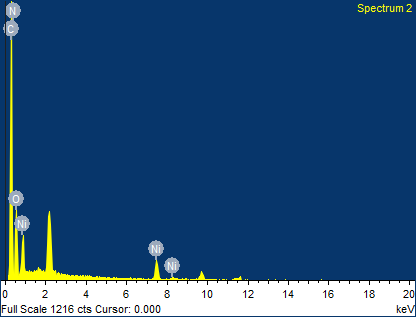

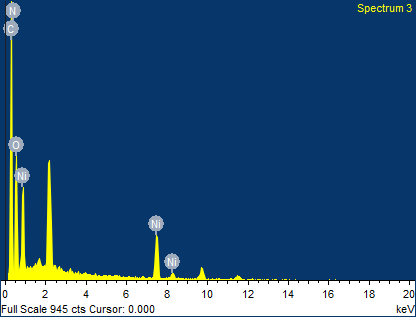


Figure 3. EDX spectra of NiNH_2_BDC MOF (a), 1wt % rGO composite (b), 2 wt % rGO composite (c), and 5 wt % rGO composite (d).
